# Quantitative Structure-Activity Relationship of Enhancers of Licochalcone A and Glabridin Release and Permeation Enhancement from Carbomer Hydrogel

**DOI:** 10.3390/pharmaceutics14020262

**Published:** 2022-01-22

**Authors:** Zhuxian Wang, Yaqi Xue, Zhaoming Zhu, Yi Hu, Quanfu Zeng, Yufan Wu, Yuan Wang, Chunyan Shen, Cuiping Jiang, Li Liu, Hongxia Zhu, Qiang Liu

**Affiliations:** School of Traditional Chinese Medicine, Southern Medical University, Guangzhou 510515, China; wangzhuxian88@smu.edu.cn (Z.W.); xyq1997@smu.edu.cn (Y.X.); zmnf1988@smu.edu.cn (Z.Z.); huyi12110357@smu.edu.cn (Y.H.); 22020286@smu.edu.cn (Q.Z.); 3161008010@smu.edu.cn (Y.W.); 521wl@smu.edu.cn (Y.W.); shenchunyan@smu.edu.cn (C.S.); jxiaqing126@smu.edu.cn (C.J.); 3188010173@i.smu.edu.cn (L.L.)

**Keywords:** carbomer hydrogel, whitening agents, enhancers, enhancement site and mechanism, drug release and permeation

## Abstract

This study aimed to systematically compare licochalcone A (LicA) and glabridin (Gla) (whitening agents) release and permeation from Carbomer 940 (CP) hydrogels with different enhancers, and evaluate the relationship between the quantitative enhancement efficacy and structures of the enhancers. An in vitro release study and an in vitro permeation experiment in solution and hydrogels using porcine skin were performed. We found that the Gla–CP hydrogel showed a higher drug release and skin retention amount than LicA–CP due to the higher solubility in medium and better miscibility with the skin of Gla than that of LicA. Enhancers with a higher molecular weight (MW) and lower polarizability showed a higher release enhancement effect (ER_release_) for both LicA and Gla. The Van der Waals forces in the drug–enhancers–CP system were negatively correlated with the drug release percent. Moreover, enhancers with a higher log P and polarizability displayed a higher retention enhancement effect in solution (ER_solution retention_) for LicA and Gla. Enhancers decreased the whole intermolecular forces indrug–enhancers-skin system, which had a linear inhibitory effect on the drug retention. Moreover, C=O of ceramide acted asthe enhancement site for drug permeation. Consequently, Transcutol^®^ P (TP) and propylene glycol (PG), seven enhancers showed a higher retention enhancement effect in hydrogel (ER_hydrogel retention_) for LicA and Gla. Taken together, the conclusions provide a strategy for reasonable utilization of enhancers and formulation optimization in topical hydrogel whitening.

## 1. Introduction

Hydrogels are used extensively in topical and transdermal drug delivery systems, among which carbomer polymers account for a significant proportion. Carbomer 940 (CP) is used in cosmetic formulation benefitting due to its moderate viscosity and good stability [1]. Drug permeation from hydrogel consists of two processes: the first is drug release from the carbomer matrix and the second is skin permeation. Previous literature has described drug–polymers interaction (mainly H–H bond interaction and Van der Waals forces) [2,3], in which the rheological properties, including the viscosity, storage modulus (G′), loss modulus (G″), and phase shift angle (δ) [4], hydration [5], and mesh size [6] of the hydrogels influence drug release. However, the most critical aspects hindering drug permeation are caused by the highly compact structure of the *Stratum corneum* (SC), which limits the efficient delivery of active pharmaceutical ingredients [7,8]. Therefore, a wide range of permeation enhancers are utilized to improve drug release or penetration from the hydrogel system.

Permeation enhancers can enhance drug release from complicated hydrogel systems by reducing the molecular mobility of the systems [9], occupying drug–polymer binding sites [3], etc. Song concluded that permeation enhancers that form hydrogen bonds, such as Span 80 (SP), weaken drug–adhesive interaction, facilitating the release of drug from adhesive [10]. On the other hand, permeation enhancers disrupt the skin lipid arrangement [11,12], change the keratin structure [13], enhance the miscibility of the skin [14], and increase drug partitioning into deeper skin layers [15] to improve the skin permeation of drugs. Plurol^®^ Oleique CC 497 (POCC) showed a preference for occupying sites where skin lipids had interacted with drugs with low PSA and polarizability due to the improved skin–POCC miscibility and stronger interactions [16]. Thus, the physicochemical properties, such as the molecular weight (MW), log P, polar surface area, and polarizability of the enhancers, determine differences in the enhancement effect. Yang systematically evaluated the enhancement action efficacy and sites of different enhancers on drug release and permeation from a patch. It was found that hydrophilic enhancers including Transcutol^®^ P (TP) and propylene glycol (PG) had a better miscibility with matrix carboxyl PSA, indicating their ability to facilitate a higher drug release. In contrast, hydrophobic enhancers including POCC and SP linked with SC lipids more easily, disrupting the lipid arrangement, and thereby improving drug permeation [17]. As a result, demonstrating the molecular interaction of drug–enhancers–CP and drug–enhancers–skin systems is significant, thus shedding new light on the reasonable utilization of enhancers in pharmaceutical and cosmetic preparations.

Licochalcone A (LicA) [18] and glabridin (Gla) [19], flavonoid compounds extracted from the roots of *Glycyrrhiza glabra* L., both have significant anti-melanogenic effects on cellular and animal levels as we previously revealed. However, the poor water solubility and higher log P hinders their transdermal permeation when applied alone, which further influences their practical use. Moreover, it was more difficult for parent LicA to permeate the skin than Gla due to its poorer water solubility and higher log P. Thus, the addition of enhancers represents an effective way to overcome these drawbacks and improve the drug absorption of the two whitening agents. It is expected that higher amounts of Gla and LicA accumulate in the epidermis and dermis rather than in the systemic circulation as melanocytes are located in the basal epidermal layers. The enhancers should firstly accomplish maximum release of the whitening agent from the matrix polymer. Consequently, the ideal enhancers must contribute to the two processes simultaneously. However, to our knowledge, few studies have focused on whitening agents’ release and skin delivery behaviors from CP hydrogel based on drug–enhancers–CP (skin) interactions, which has resulted in blindness and uncertainty regarding the utilization of enhancers in whitening formula optimization. Meanwhile, no investigations have systematically compared the enhancing effect of Gla and LicA by different enhancers.

**Figure 1:**
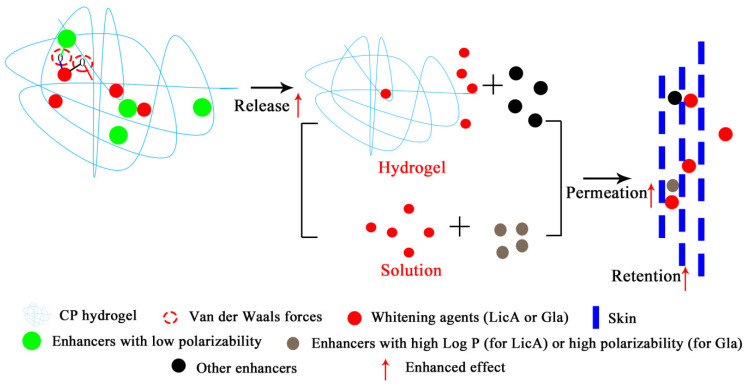
Schematic showing Gla and LicA release and permeation from CP hydrogel with enhancers.

Therefore, for the first time, this study systematically reported the quantitative enhancement efficacy and site of action explaining the drug release and skin absorption of LicA and Gla (whitening agents) from CP hydrogel by different enhancers (Figure 1). Seven enhancers, including POCC, TP, PG, SP, Capryol™ 90 (CP 90), N-methylprolinodone (NMP),and isopropyl myristate (IPM) with different physicochemical parameters (Table 1), were selected. Firstly, Gla–CP and LicA–CP hydrogels with or without enhancers were prepared. The drug release behavior from different CP hydrogels was evaluated by an in vitro drug release experiment. The drug release enhancement effect (ER_release_) and interaction parameters of Gla (LicA)–CP and Gla (LicA)–enhancers–CP hydrogels were demonstrated next. Then, the porcine skin was used to evaluate the enhanced retention and permeation effect of Gla and LicA in solution (ER_solution retention_, ER_permeation_) and hydrogel (ER_hydrogel retention_, ER_com_) by enhancers, followed by the enhancement site and mechanisms involved in it. In addition, the correlation between the drug release amount, drug permeation amount, drug retention amount and physicochemical parameters of the enhancers (Table 1), energy of mixing (E_mix_), and cohesive energy density (CED) were investigated, respectively. These results provide insight into the drug–enhancers–CP and drug–enhancers–skin interactions and the structural characteristics of enhancers, which lays a solid basis for the drug-specific molecular mechanisms of enhancers and pharmaceutical hydrogel design. Moreover, it predicted information for the topical application of enhancers with specific structures for high drug release and skin retention of whitening formulation.

## 2. Materials and Methods

### 2.1. Material

Licochalcone A (LicA, purity >98%) and glabridin (Gla, purity >98%) were obtained from Nanjing Spring & Autumn Biological Engineering Co., Ltd. (Nanjing, China). Poly(acrylic acid) (commercial names: Carbomer 940 (CP)), isopropyl myristate (IPM, purity: 98%), diethylene glycol monoethyl ether (commercial names: Transcutol^®^ P (TP), purity: 99%), and 1-methyl-2-pyrrolidinone (NMP, purity >99%) were purchased from Shanghai Macklin Biochemical Co., Ltd. (Shanghai, China). Propylene glycol monocaprylate (commercial names: Capryol^TM^ 90 (CP 90)) and polyglyceryl-3dioleate (commercial names: Plurol^®^ Oleique CC 497 (POCC)) were supplied by Gattefossé (Lyon, France). Polyoxyethylenesorbitan monooleate (commercial names: Span 80 (SP)) and propylene glycol (PG, purity > 99%) were purchased from Damao Chemical Reagent Factory (Tianjin, China). Polyethylene glycol 400 (PEG 400) and cellophane membranes were purchased from Beijing Solarbio Technology Co., Ltd., Beijing, China. All other reagents were analytical grade.

### 2.2. Preparation of Hydrogels

The LicA–CP and Gla–CP hydrogels (cargo loading: 5%, *w/w*) were prepared as follows: First, 2 g of CP were dispersed in 100 mL of deionized water and stored at room temperature for 24 h to fully swell the hydrogels. Subsequently, LicA and Gla dissolved in ethanol were added to the CP hydrogels, and the mixture was stirred until it was homogeneous. The pH was to 5 with NaOH solution. Hydrogels with enhancers (drug–enhancers–CP) (cargo loading: 10%, *w/w*) were also fabricated using the same methods. Hydrogels were stored at 4 °C and hydrogel films were prepared using the freeze-dried technique.

### 2.3. Rheological Properties of Hydrogel

The rheological tests were performed in a MARS iQ Air + P35 rheometer (Thermo Scientific HAAKE, New York, NY, USA).The flow properties of the CP, LicA–CP, and Gla–CP hydrogels were obtained from continuous shear flow tests with shear rates ranging from 0–120 s^−1^ for 120 s. Continuous ramp step were selected for analysis. The elastic and viscous modulus at different frequencies was measured using frequency sweep tests ranging from 0.1 to 100 with a constant %strain of 0.1. The G′, G′′, and δ values were recorded and the frequency was plotted in a logarithmic scale.

### 2.4. Determination of the Drug Solubility in the Donor Phase

Briefly, excessive Gla or LicA was added to the PBS/PEG400 (*v/v*, 80/20) and subjected to ultrasound for 30 min at 25 °C. Subsequently, the supernatant of the solution was taken out and filtered with a 0.22 μm microporous membrane for HPLC analysis. The detailed HPLC methods are shown in Methods S1.

### 2.5. In Vitro Release of Hydrogels

The hydrogels (0.3 g) were added to the cellophane membranes, which were then fixed between the donor and the receptor cells of the Franz diffusion cells (effective diffusional area: 1.77 cm^2^; volume: 15 mL; TP-6, China). PBS (pH = 7.4) containing 20% PEG 400 (*v/v*) was chosen as the acceptor medium and stirred at 350 rpm and 32 °C. In total, 1.0 mL of sample was withdrawn after 0.25, 0.5, 1, 2, 3, 4, 6, 8, 10, 12, 24, 36, and 48 h and then replaced by the same volume of fresh medium. The samples were analyzed using HPLC (Agilent 1260, Santa Clara, CA, USA). The detailed HPLC methods are provided in Methods S1.

R_hydrogel_ and Q_hydrogel_ represent the cumulative release percent of drug (%) and cumulative release amount of drug, respectively. The enhancement ratio of drug release in hydrogel (ER_release_) was calculated as follows:

(1) $${\mathrm{ER}}_{\mathrm{release}}=\frac{R_{\mathrm{hydrogel}\;\mathrm{with}\;\mathrm{enhancer}}}{R_{\mathrm{hydrogel}\;\mathrm{without}\;\mathrm{enhancer}}}$$

### 2.6. Mechanism of Enhancement Drug Release

#### 2.6.1. Attenuated Total Reflection FT-IR (ATR-FT-IR) of the CP Hydrogels

The ATR-FT-IR study was used to confirm the effect of the enhancers on the drug release of the CP systems. The hydrogel films were obtained from the preparation of hydrogels, and the infrared spectra were recorded using a Nicolet iS50 FT-IR spectrometer (Thermos, New York, NY, USA) within the frequency range of 500–4000 cm^−1^ using 32 scans at a resolution of 2.

#### 2.6.2. Raman Spectroscopy

Raman spectra were also used to characterize the potential interactions among the drug, enhancers, and CP by a Raman spectrometer (Renishaw RM2000, London, England). Then, the samples were measured at 25 °C using a 785 nm laser source with 500 mW power.

#### 2.6.3. X-ray Diffraction (XRD)

The proportion of crystalline in different hydrogels was determined by diffracted intensity measurement using an X-ray diffractometer (SmartLab 3KW, Rigaku, Japan) of Cu Kα radiation in the 5–60° 2θ range with a scan rate of 10°/min.

#### 2.6.4. Polarized Light Microscopy (PLM)

PLM measurement was conducted to confirm the results of XRD using a Nikon polarized optical microscope (edipse lv100N pol, Tokyo, Japan). The images were captured using QImaging software (Nis-Elements F) with a first-order compensator at 100× magnification.

#### 2.6.5. Differential Scanning Calorimetry (DSC)

The hydrogel films were placed in the aluminum DSC pans of a thermal analyzer (TA Q2000, TA, New Castle, Lindon, UT, USA), and then heated from 25–250 °C at a rate of 10 °C/min with 3 cycles (to eliminate the thermal history). All samples were performed under a nitrogen atmosphere (40 mL/min). The parameter glass transition temperature (T_g_) was recorded at the midpoint of the transition in the curve.

#### 2.6.6. Molecular Interaction Study: Molecular Docking

Molecular docking was conducted to corroborate the results of FT-IR to calculate the intermolecular strength of the Gla (LicA)–CP and Gla (LicA)–enhancers–CP systems using Materials Studio version 8.0 (Accelrys, San Diego, CA, USA). The molecular structures of the CP, Gla, LicA, and seven kinds of enhancers were obtained from the PubChem database and subjected to geometry optimization with Forcite modules in the COMPASS II force field. Next, the mixing energy (E_mix_) and interaction parameters (χ) were calculated. In addition, the optimized structure of the Gla (LicA)–CP and Gla (LicA)–enhancers–CP associations were obtained.

#### 2.6.7. Molecular Dynamic Simulation

Molecular dynamic simulation was utilized to understand the drug release behaviors of different hydrogels with or without enhancers. The optimized CP, Gla (LicA), and enhancers were placed in the amorphous cell modules according to the proportions of the actual formulation, and the built systems were further optimized by For cite modules. Subsequently, NVT equilibration of 50 ps at 298 K was conducted for each system, after which NPT equilibration of 100 ps was performed at 305 K and 101.325 Kpa with a time step of 1 ps. The CED was calculated for each system. Then, snapshots of the hydrogel systems at the end of the MD were obtained.

### 2.7. Correlation Analysis 1

First, a linear regression analysis was conducted to investigate the relationship between the drug release amount and physicochemical parameters, including MW, log P, polarizability, and polar surface area, in different hydrogels using SPSS 20.0 software (SPSS, Chicago, IL, USA). On the other hand, the linear regression equation of the drug release amount and E_mix_ and CED were calculated.

### 2.8. In Vitro Skin Permeation of Drug Solution and Hydrogel

Porcine skin (one-month-old Bama miniature pig, male, 20 kg) was supplied by Aperture Biotech Co., Ltd. (Hong Kong, China) The thickness of porcine skin was maintained at approximately 800 μm and its structural integrity was guaranteed before the experiments. The porcine skin sample was sandwiched between the donors and receptors compartment in Franz diffusion cells with the dermal side facing downwards. Then, different drug aqueous solutions (0.3 g) and 0.3 g of the corresponding hydrogels were added to the donor receptors, and PBS/PEG400 (*v/v*, 80/20) was chosen as the medium to obtain sink conditions. In total, 1 mL of receptor vehicle was withdrawn after1, 2, 4, 6, 8, 10, 12, 24, 36, and 48 h. Others were processed similar to the in vitro release of the hydrogel. All animal experiments were performed in accordance with the “Guiding Principles in the Care and Use of Animals” (China), and approved by the Ethics Committee of Southern Medical University (L2019036, date of approval: 13 April 2019).

P_solution_ and P_hydrogel_ represent the cumulative permeation in the solution and hydrogel, respectively.

The enhancement ratio of the drug skin permeation in the drug solution (ER_permeation_) was calculated as follows:

(2) $${\mathrm{ER}}_{\mathrm{permeation}}=\frac{P_{\mathrm{solution}\;\mathrm{with}\;\mathrm{enhancer}}}{P_{\mathrm{solution}\;\mathrm{without}\;\mathrm{enhancer}}}$$

The enhancement ratio of the drug skin permeation in hydrogel (ER_com_) was calculated as follows:

(3) $${\mathrm{ER}}_{\mathrm{com}}=\frac{P_{\mathrm{hydrogel}\;\mathrm{with}\;\mathrm{enhancer}}}{P_{\mathrm{hydrogel}\;\mathrm{without}\;\mathrm{enhancer}}}$$

β_R/P_ was calculated to evaluate the sites of action of the enhancers [17]:

(4) $$\beta_{R/P}=\frac{{\mathrm{ER}}_{\mathrm{release}}}{{\mathrm{ER}}_{\mathrm{permeation}}}$$

The rate-limiting step of transdermal drug delivery was assessed using the following equation:

(5) $$F=\frac{P_{\mathrm{hydrogel}}}{Q_{\mathrm{hydrogel}}}$$

### 2.9. Drug Retention

After in vitro skin permeation, the treated skin samples were removed from the diffusion cell. Subsequently, the skin at the administration site was wiped to remove the unabsorbed drug, cut into pieces, weighed, and extracted with methanol by ultrasound for 1 h. Then, the supernatant was detected with HPLC to obtain the skin retention amount.

RE_solution_ and RE_hydrogel_ are the cumulative retention amount of drug in the solution and hydrogel, respectively.

The enhancement ratio of the drug skin retention in solution (ER_solution retention_) was calculated as follows:

(6) $${\mathrm{ER}}_{\mathrm{solution}\;\mathrm{retention}}=\frac{{\mathrm{RE}}_{\mathrm{solution}\;\mathrm{with}\;\mathrm{enhancer}}}{{\mathrm{RE}}_{\mathrm{solution}\;\mathrm{without}\;\mathrm{enhancer}}}$$

The enhancement ratio of the drug skin retention in hydrogel (ER_hydrogel retention_) was calculated as follows:

(7) $${\mathrm{ER}}_{\mathrm{hydrogel}\;\mathrm{retention}}=\frac{{\mathrm{RE}}_{\mathrm{hydrogel}\;\mathrm{with}\;\mathrm{enhancer}}}{{\mathrm{RE}}_{\mathrm{hydrogel}\;\mathrm{without}\;\mathrm{enhancer}}}$$

### 2.10. Mechanism of Enhancement Drug Permeation

#### 2.10.1. ATR-FT-IR Spectra of the Porcine Skin

The ATR-FT-IR study was used to investigate the effect of the enhancers on the arrangement variations of the skin lipid and protein region. The skin samples were taken from the in vitro skin permeation of drug solution, and the infrared spectra were recorded as described in Section 2.6.1.

#### 2.10.2. Confocal Laser Microscope (CLSM)

CLSM was used to visualize the LicA and Gla distribution in the skin tissue, and C6 was utilized as a substitute for Gla. Treated skin samples were processed similar to the in vitro skin permeation of drug solution with a permeation time of8 h. The samples were cut longitudinally into 6-μm-thick slices using a Cryostat microtome (Thermo HM525 NX, New York, NY, USA) after fixation. LicA and C6 were emitted at 480 and 485 nm using a confocal laser microscope (CLSM 800, ZEISS, Jena, Germany), respectively.

#### 2.10.3. Molecular Docking and Molecular Dynamic Simulation

Ceramide 2 was used as are presentative of skin lipids for molecular docking and molecular dynamic simulation due to it having the highest proportion in skin lipids [14,20]. The E_mix_, χ, and CED of Gla (LicA)-skin and Gla (LicA)–enhancers-skin systems and their snapshots were obtained as described.

### 2.11. Correlation Analysis 2

A multiple linear regression model was also used to detect the correlation between the drug retention, drug permeation amount, and physicochemical parameters of the enhancers as described before. Moreover, the relationships between the drug retention or drug permeation amount and C=O band displacement value in the FT-IR, E_mix_, and CED were calculated.

### 2.12. Statistical Analysis

All data were analyzed using SPSS 20.0 software (Chicago, IL, USA). Data were expressed as mean ± SD and subjected to one-way analysis of variance (ANOVA) or two-tailed paired Student’s *t*-test. The significance level was set at *p* < 0.05.

## 3. Results

### 3.1. Preparation of the CP–Gla and CP–LicA Hydrogel

**Figure 2:**
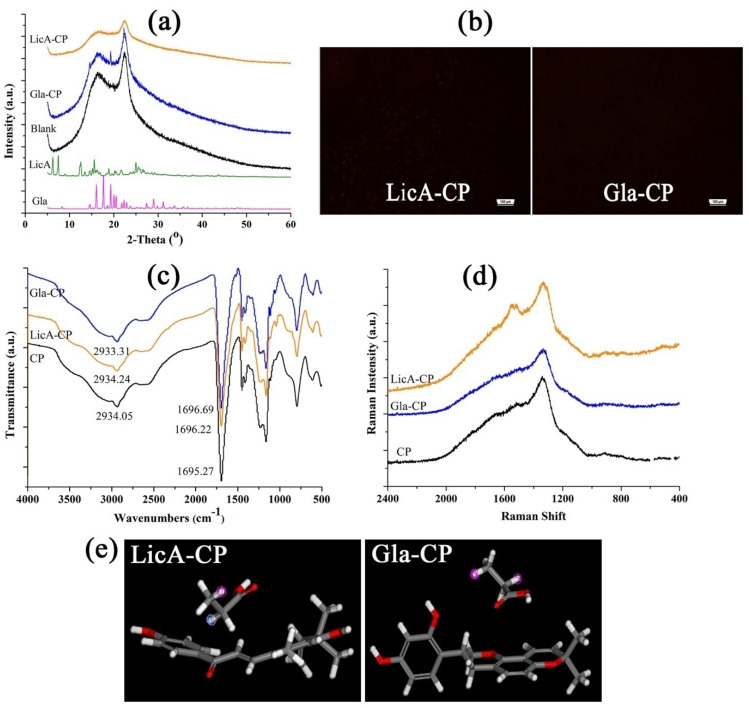
(**a**) X-ray powder diffractograms of different hydrogels; (**b**) PLM images of CP films; (**c**) FT-IR spectra of CP, LicA–CP, and Gla–CP; (**d**) Raman spectra of the hydrogels; (**e**) conformations of LicA-CP and Gla-CP.

Lyophilized hydrogels were prepared to investigate the potential interaction between the drugs and CP. First, XRD (Figure 2a) and PLM analyses (Figure 2b) were used to detect the crystals in the hydrogel films, and the results demonstrated that both Gla and LicA almost completely dissolved in the CP hydrogel without the formation of obvious crystals. Moreover, LicA and Gla displayed a similar miscibility to CP. These results indicate that Gla and LicA were molecularly dispersed in the hydrogel, which laid a foundation for the hydrogen bond or the formation of other interactions [21]. The hydroxyl (−OH) and carbonyl group (C=O) of Gla and LicA, and the carboxyl (−COOH) group of CP are the functional groups that may potentially be involved in the drug–CP interaction. In the blank CP, the characteristic band at 2934.05 cm^−1^ was assigned to –OH stretching vibration while the band at 1695.27cm^−1^ was attributed to C=O stretching of the CP (Figure 2c). The band at 2934.05 cm^−1^ shifted to 2933.31 and 2934.24 cm^−1^, and the C=O band moved to 1696.69 and 1696.22 cm^−1^ for Gla-CP and LicA-CP, respectively, indicating weak interaction in the drug–CP systems. Furthermore, the Gla–CP system showed a stronger interaction strength than LicA-CP. The Raman spectra (Figure 2d) also confirmed the presence of the interaction due to the movement of the −OH band. The values of E_mix_ and χ measured using molecular docking are used to estimate the strength of the intermolecular interactions. The closer E_mix_ and χ are to 0, the greater the miscibility and the stronger the intermolecular interactions. In this case, the Gla–CP system possessed a lower E_mix_ and χ than LicA–CP, further underscoring the stronger interaction between Gla and CP (Table 2). The optimized structures of the Gla (LicA)–CP binary associations are displayed in Figure 2e.

### 3.2. In Vitro Release of Gla–CP and LicA–CP Hydrogels

**Figure 3:**
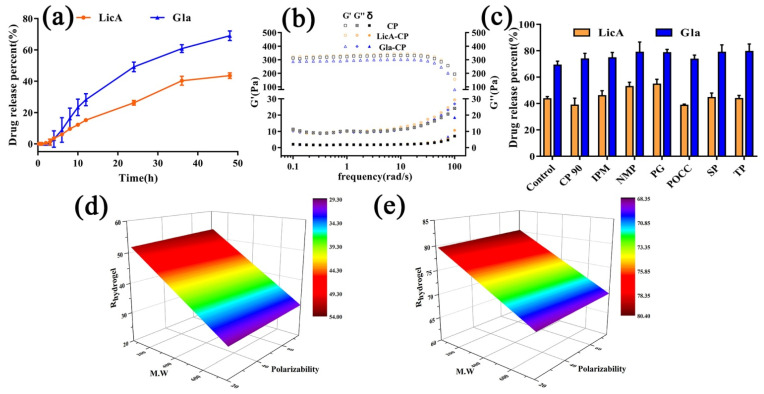
(**a**) In vitro drug release profiles of hydrogel (*n* = 3); (**b**) frequency sweep (G′, G″, and δ) of the hydrogel (*n* = 3); (**c**) LicA and Gla release percent after48 h after different enhancers were added (*n* = 3); (**d**) response surface plot demonstrating the effect of MW and polarizability on the ERrelease of LicA; (**e**) response surface plot demonstrating the effect of MW and polarizability on the ER_release_ of Gla.

The results (Figure 3a) showed that Gla displayed a higher release amount and release rate than LicA, indicating that the interaction strength in the drug–CP systems was not a dominating factor controlling the drug release. However, the highest release percent of Gla and LicA only reached 69.08% and 43.56%, respectively, after 48 h. Moreover, the release behaviors of Gla and LicA from CP hydrogel followed the zero-order equation, and the release equations of the release amount percent and time are listed as follows:

R_hydrogel_ (LicA) = 1.01 × t + 0.20 (R^2^ = 0.97) (8)

R_hydrogel_ (Gla) = 1.61 × t + 0.70 (R^2^ = 0.95) (9)

To demonstrate the influencing effect on the drug release, DSC study reflecting the free volume and mesh size of the hydrogels was carried out. The results (Figure S1a) demonstrated that Gla–CP showed a similar T_g_ to LicA–CP, which is indicative of a slight effect of the mesh size on the drug release. In addition, G′ represents the rigidity of hydrogels [22,23], and G″ is a parameter used to demonstrate the friction of a molecular chain and reflect changes in the intermolecular interaction [24]. The rheological study (Figure 3b and Figure S1b) revealed that the zero-shear viscosity, G′, G″, and δ of Gla–CP all showed no significant difference to that of LicA–CP, indicating that the viscoelastic properties of the hydrogel also had no significant influence on the drug release.

### 3.3. In Vitro Release of Drug in the Presence of Enhancers

To improve the drug release from the hydrogels, different enhancers were added to hydrogels and in vitro release experiments were performed (Figure S1c,d). We found that only PG (highest ER_release_: 1.25) and NMP significantly increased the LicA release percent while TP (highest ER_release_: 1.15), POCC, SP, and IPM all contributed to a significantly higher Gla release percent (Figure 3c and Table 3) after 48 h. Then, multivariate linear regression analysis was conducted to investigate the effect of the physicochemical parameters of the enhancers on R_hydrogel_. The regression equations areexpressed as follows:

R_hydrogel_ (LicA) = 52.87 + 0.057 × M.W − 0.74 × Polarizability (10)

R_hydrogel_ (Gla) = 79.76 + 0.03 × M.W − 0.36 × Polarizability (11)

The response surface plot (Figure 3d,e) showed that both R_hydrogel_ of Gla and LicA were negatively correlated with the polarizability, and positively correlated with MW of the enhancers. Moreover, the polarizability dominated the drug release. Polarizability represents the ability of Van der Waals forces to form [25], which are the primary interaction forms in drug–enhancers–CP systems. Enhancers with higher polarizability tended to be linked with drug–CP systems, which is a sign of stronger intermolecular interactions forming in the drug–enhancers–CP ternary systems, thereby decreasing the drug release percent. These results prove the interaction strength in drug–enhancers–CP systems was an important factor determining the drug release.

### 3.4. Molecular Modeling and Correlation Analysis 1

Then, E_mix_ and χ of different Gla (LicA)–enhancers–CP systems were calculated (Table 2) using Materials Studio version 8.0, and their optimized ternary associations are displayed at Figure S2. For LicA–enhancers–CP, LicA–PG–CP hydrogel showed the highest χ (19.48) and E_mix_ (11.54) while LicA–SP–CP showedthe lowest (−1.77 and −1.05). For Gla–enhancers–CP, the Gla–PG–CP system showed the worst miscibility, with χ of 8.85 and E_mix_ of 5.24, whereas POCC showed the best miscibility with Gla–CP. Linear regression of the drug release percent and E_mix_ was performed to clarify the effect of the interaction strength on R_hydrogel_. The linear regression equation of R_hydrogel_ and E_mix_ is as follows:

R_hydrogel_ (LicA) = 0.92 × E_mix_ + 42.10 (R^2^ = 0.57) (12)

R_hydrogel_ (Gla) = 1.64 × E_mix_ + 71.75 (R^2^ = 0.82) (13)

**Figure 4:**
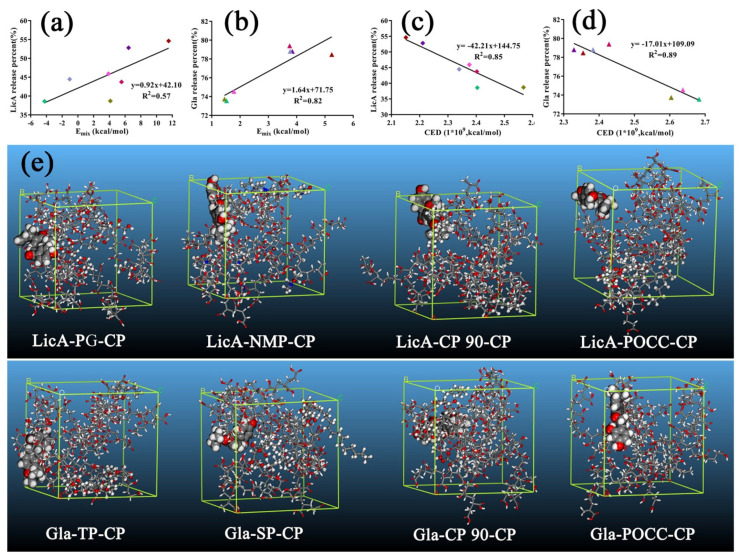
(**a**) The correlation relationship between the LicA release percent and E_mix_ of ternary systems; (**b**) linear analysis of the Gla release percent and E_mix_ of ternary systems; (**c**) the correlation relationship between the LicA release percent and CED of ternary systems; (**d**) linear analysis of the Gla release percent and CED of ternary systems; (**e**) snapshots of the LicA (Gla)–enhancers–CP systems at the end of the MD (drug: ball and stick model; enhancers: CPK model).

The equations (Figure 4a,b) showed that the better the compatibility between the enhancers and drug–CP binary systems, the lower the release amount. These findings are consistent with the above results. To further corroborate the effect of intermolecular forces on the drug release, molecular dynamics simulation was carried out to calculate the CED values to reflect the interactions among the drugs, enhancers, and CP. The results are displayed in Table 2 and snapshots of the hydrogels systems at the end of the MD are shown in Figure 4e and Figure S3. A higher CED value means a stronger interaction [3]. Similarly, linear regression of the drug release percent and CED was also conducted, and the linear regression equations are expressed as follows:

R_hydrogel_ (LicA) = −42.21 × CED + 144.75 (R^2^ = 0.85) (14)

R_hydrogel_ (Gla) = −17.01 × CED + 119.09 (R^2^ = 0.89) (15)

The results (Figure 4c,d) further emphasize the decreasing effect of Van der Waals forces on the drug release. The stronger the interaction strength in the compound systems, the lower the drug release percent.

### 3.5. The Release Mechanism of the Drug from the Drug–Enhancers–CP System

**Figure 5:**
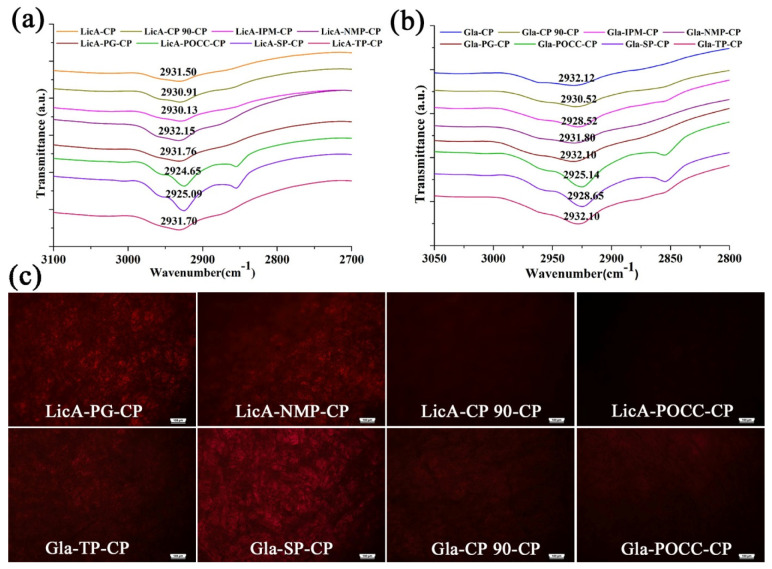
(**a**) FT-IR spectra (−OH group) of LicA–enhancers–CP systems; (**b**) FT-IR spectra (−OH group) of Gla–enhancers–CP systems; (**c**) PLM images of drug–CP films after different enhancers were added.

To demonstrate the drug release mechanism of the ternary systems, FT-IR and PLM were conducted. LicA–CP was used as the control group, and CP showed a typical band at 2931.5 cm^−1^ representing the –OH group (Figure 5a,b), while the band at 1696.21 cm^−1^ belonged to the C=O band (Figure S4a and Figure 4b). Upon mixing SP or POCC with CP, the −OH bands showed a red shift to 2925.14 and 2924.66 cm^−1^, respectively (Figure 5a), attributing to a strong interaction between SP or POCC and CP. However, the position of the −OH band did not show any significant difference when mixing CP 90 or NMP with CP. Moreover, only NMP and POCC induced a weak movement of the C=O band for LicA–CP (Figure S4a). For the Gla–CP binary systems, the bands appearing at 2932.12 and 1696.6 cm^−^^1^ also represented the −OH and C=O groups, respectively. The addition of SP or POCC also led to significant movement of the−OH band (Figure 5b), which was similar to LicA–CP. In contrast, upon loading with NMP and IPM, the −OH band showed no significant difference with the control group. The bands of the C=O groups did not show significant movement except for the addition of NMP (Figure S4b). The results revealed that the –OH of CP was not the enhancement site for LicA and Gla release.

PLM was used to observe the re-crystallization of the drug after the addition of enhancers. Higher drug re-crystallization indicates weaker enhancers–CP interactions and a better drug release ability. After mixing with SP, CP 90, IPM, NMP, or POCC, no significant LicA crystals were detected in the drug–enhancers–CP films (Figure 5c and Figure S4c). However, a significantly larger amount of LicA crystals were observed in the film system after PG or NMP addition. These results indicated that the enhancers, such as PG or NMP, occupied the LicA–CP binding site, enabling LicA release from the hydrogel. For the Gla–CP system, upon adding NMP, PG, SP, or TP, a significantly larger amount of Gla crystals appeared in the hydrogel film. In contrast, IPM, CP 90, and POCC showed better miscibility with the Gla–CP systems; therefore, no Gla was detected in these ternary systems. These results are consistent with the in vitro release study.

### 3.6. In Vitro Skin Permeation and Drug Retention of Drug Solution

**Figure 6:**
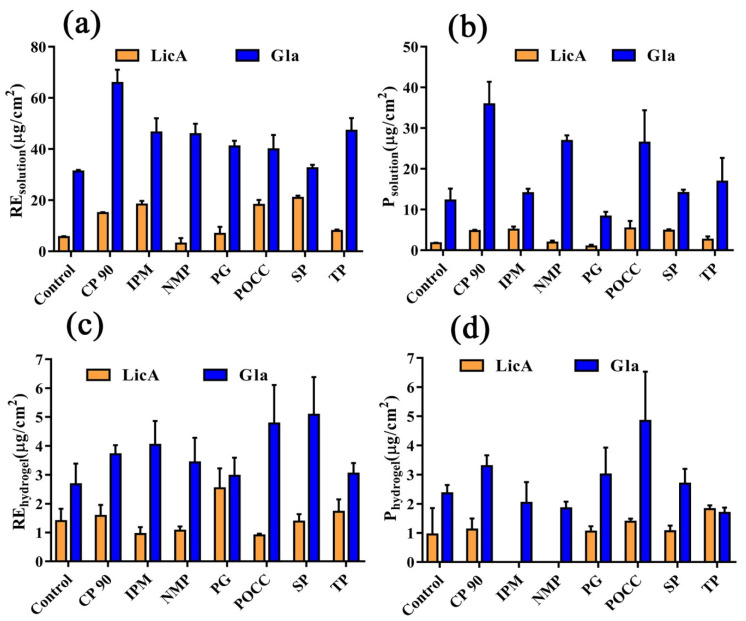
(**a**) RE_solution_ of LicA and Gla after 48 h (*n* = 4); (**b**) P_solution_ of LicA and Gla after 48 h (*n* = 4); (**c**) RE_hydrogel_ of LicA and Gla after 48 h (*n* = 4); (**d**) P_hydrogel_ of LicA and Gla after 48 h (*n* = 4).

A comparison of the enhancement of LicA and Gla skin retention after 48 h is shown in Figure 6. The results demonstrated that the amount of Gla that accumulated in the skin within 48 h was 5.63 times higher than that of LicA (Figure 6a). Furthermore, approximately a 7.02 times increase in the amount of Gla permeating into the receptor fluids was observed when compared with LicA (Figure 6b). The addition of CP 90, POCC, SP, and IPM significantly enhanced the retention of LicAin the skin, and the enhancement effect was rank ordered as SP (ER_solution retention_: 3.78) > POCC ≈ IPM > CP 90 > TP > PG > NMP (Figure 6a and Table 3). CP 90, POCC, SP, and IPM also significantly facilitated LicA’s permeation across the skin and POCC showed the highest ER_permeation_ value (Figure 6b and Table 3). However, the seven enhancers all significantly improved Gla’s disposition into the skin, and the enhancement effect followed the order of CP 90 (ER_solution retention_: 2.11) >TP ≈ IPM≈ NPM >PG > POCC > SP (Figure 6a and Table 3). However, only POCC, CP 90, and NMP significantly facilitated Gla’s permeation and CP 90 showed the highest ER_permeation_value (Figure 6b and Table 3). In addition, it was observed that the permeation amount of LicA and Gla showed a positive linear relation with the LicA and Gla retention amount (R^2^ = 0.85 and R^2^ = 0.47), respectively, indicating that the seven enhancers all demonstrated a similar contributory effect on the drug retention and drug permeation.

### 3.7. The Enhancement Mechanism of the LicA and Gla

#### 3.7.1. ATR-FT-IR of the Skin

**Figure 7:**
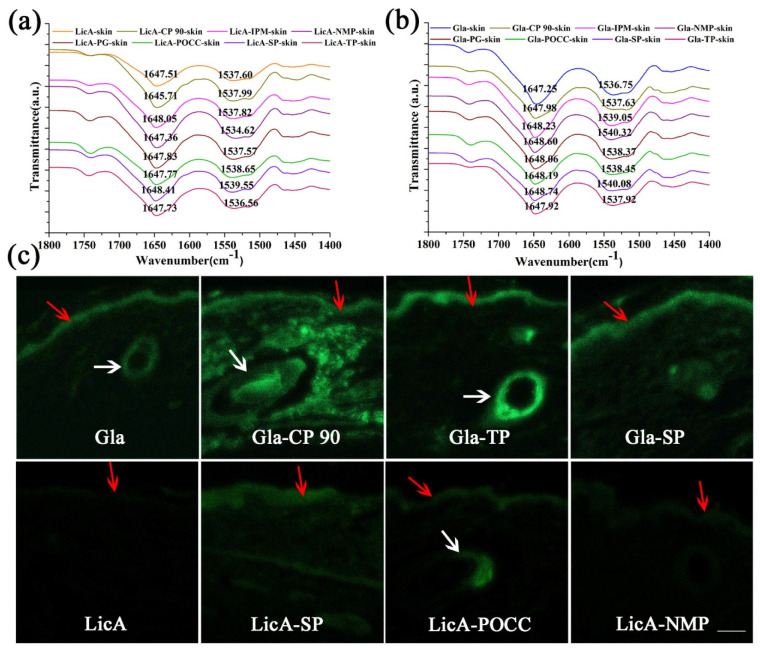
(**a**) FT-IR spectra (C=O group) of LicA–enhancers–skin systems; (**b**) FT-IR spectra (C=O group) of Gla–enhancers–skin systems; (**c**) CLSM images of the penetration depth and fluorescence intensity LicA and C6 in porcine skin treated with enhancers (Bar = 100 μm, the red arrows and white arrows represent the SC and hair follicles of the skin, respectively).

ATR–FT-IR was conducted to elucidate the effects of the enhancers on the lipid and keratin arrangement of the porcine skin, and to further characterize drug–enhancers–skin interactions. The characteristic infrared absorption bands at 2918.02 and 2850.44 cm^−1^ represent the asymmetric V_as_CH_2_ and symmetric V_s_CH_2_ stretching vibrations of SC lipid (Figure S5a,b), and the bands at 1647.97 and 1538.07 cm^−1^ correspond to Amide I and Amide II of keratin (Figure 7a,b). In the LicA–skin control group, when POCC was added, the V_as_CH_2,_ V_s_CH_2_, and Amide II moved to 2920.05, 2851.60, and 1538.65 cm^−1^, respectively (Figure S5a). SP also caused a blueshift of the Amide I and Amide II bands to 1648.41 and 1539.55 cm^−1^, respectively (Figure 7a). The results indicate that POCC and SP interacted with keratin of SC and disrupted the protein structure for enhanced drug permeation and retention. However, PG and NMP did not induce any changes in the lipids and keratin bands, suggesting an insignificant enhancement of LicA retention. When Gla–skin was considered as an entirety, the seven enhancers all changed the Amide I and Amide II bands to a higher position (blueshift) to different degrees (Figure 7b), indicating that the enhancers promoted Gla deposition by changing the secondary structures of the proteins. However, no linear correlation between the drug retention amount and the Amide I and Amide II bands’ displacement values was observed. These results prove that the C=O group of porcine skin was the main enhancement site for LicA and Gla permeation.

#### 3.7.2. CLSM

The results of CLSM are shown in Figure 7c and Figure S5c. Coumarin 6 was used as a probe as a substitute for Gla in this study. It was observed that LicA permeated to a deeper skin layer in the presence of CP 90, POCC, SP, and IPM, and significantly stronger LicA fluorescence was distributed in the epidermis and dermis when compared with the parent LicA. However, PG, TP, and NMP did not facilitate drug permeation into deeper skin layers. For Gla, the seven enhancers all improved the drug retention amount and drug fluorescence intensity, and CP 90 and TP had the most significant improvement effect. The results are in accordance with the in vitro skin permeation and retention study. Interestingly, we found that hair follicles were the main permeation routes for LicA and Gla, and most fluorescence was located in the hair follicles.

#### 3.7.3. Molecular Modeling and Correlation Analysis 2

**Figure 8:**
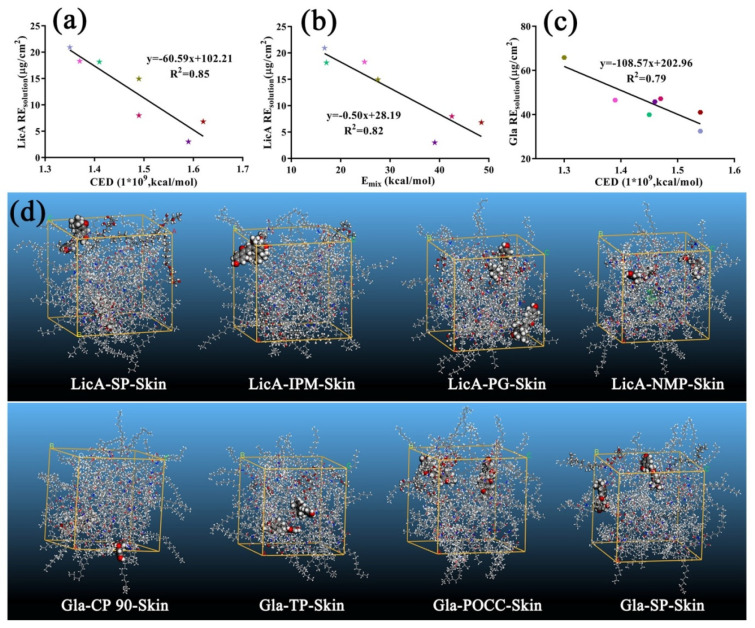
(**a**) Correlation analysis between LicARE_solution_ and CED of ternary systems; (**b**) correlation analysis between LicARE_solution_ and E_mix_ of ternary systems; (**c**) the correlation relationship between GlaRE_solution_ and CED of ternary systems; (**d**) snapshots of LicA(Gla)–enhancers–skin systems at the end of the MD (drug: ball and stick model; enhancers: CPK model).

Gla showed a higher E_mix_ (12.24) with skin than that of LicA (9.96), indicating that LicA showed better miscibility with skin than that of Gla. However, CED of Gla–skin was similar to LicA–skin. Then, the E_mix_, χ and CED values of different drug–enhancers–skin ternary systems were calculated as before (Table 4).The optimized ternary associations are displayed in Figure 8d, Figures S6 and S7. After enhancers were added, they could occupy the site of drug–skin interaction and link with the skin, leading to better compatibility between the enhancers and skin. Thus, a lower E_mix_ value is indicative of better enhancers–skin interaction and higher drug permeation [17].

Next, multivariate linear regression analysis was conducted to confirm the correlation between the P_solution_, RE_solution_, and physicochemical parameters of enhancers and the regression equations are expressed as follows:

P_solution_ (LicA) = 1.90 + 7.59 × log P (R^2^ = 0.88) (16)

RE_solution_ (LicA) = −2.26 + 0.49 × log P (R^2^ = 0.89) (17)

P_solution_ (Gla) = −1.57 + 3.84 × Polarizability − 9.12 × log P (18)

RE_solution_ (Gla) = 34.90 − 0.34 × M.W + 3.85 × Polarizability (19)

**Figure 9:**
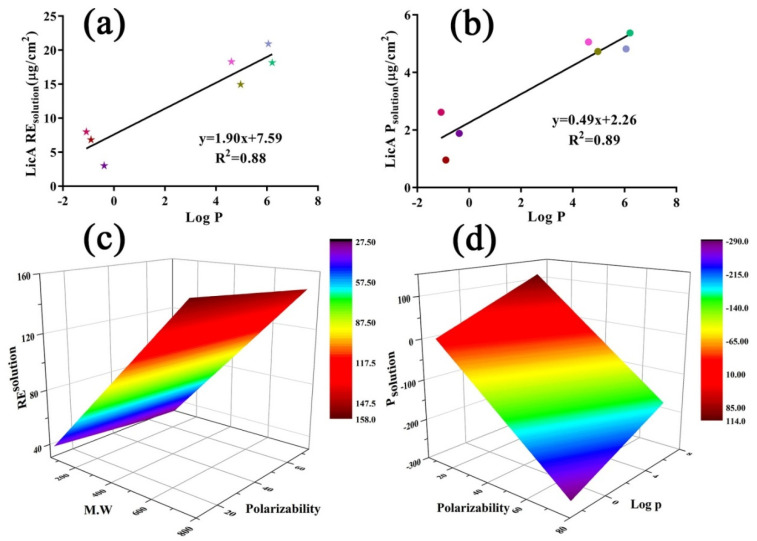
(**a**) The correlation relationship between LicARE_solution_ and log P of enhancers; (**b**) linear analysis of LicAP_solution_ and log P of enhancers; (**c**) response surface plot demonstrating the effect of MW and polarizability on RE_solution_ of Gla; (**d**) response surface plot demonstrating the effect of log P and polarizability on P_solution_ of Gla.

The results (Figure 9a,b) showed that both RE_solution_ and P_solution_ of LicA were positively correlated with log P of the enhancers. The response surface plots (Figure 9c,d) showed that RE_solution_ and P_solution_ of Gla increased as the polarizability increased. A linear regression of the drug retention amount and E_mix_ or CED was also carried out to explain the interaction force on drug retention, respectively, and the linear regression equations are expressed as follows:

RE_solution_ (LicA) = −60.59 × CED + 102.21 (R^2^ = 0.85) (20)

RE_solution_ (LicA) = −0.50 × E_mix_ + 28.19 (R^2^ = 0.82) (21)

RE_solution_ (Gla) = −108.57 × CED + 202.96 (R^2^ = 0.79) (22)

The results (Figure 8a,b) indicated that both the retention of LicA and Gla increased as the CED or E_mix_ value decreased, revealing the inhibitory effect of the intermolecular force on the drug retention. Taken together, for LicA–skin, enhancers with a higher log P showed better miscibility with skin, which resulted in increased LicA retention in the skin. For the Gla–skin binary system (Figure 8c), enhancers with a higher polarizability tended to occupy the site of the skin, thereby facilitating greater Gla deposition on the skin.

### 3.8. In Vitro Skin Permeation and Drug Retention of Drug Hydrogel

For the hydrogel system, only PG and TP could facilitate a significantly higher amount of LicA accumulation in the skin, and PG possessed the highest ER_hydrogel retention_ value. The seven enhancers all improved Gla retention in the CP systems, and the ER_hydrogel retention_ value was ranked as SP > POCC > IPM > CP > NMP > TP > PG (Figure 6c and Table 3). Moreover, a significantly higher amount of LicA was detected in the diffusion cells from hydrogel after the intervention of TP and POCC. Furthermore, only CP 90 and POCC could significantly disrupt the skin barrier for Gla penetration from the hydrogel (Figure 6d). β_R/P_ values >1 indicate that the enhancers mainly facilitated the drug release process while β_R/P_ values <1 indicate that the enhancement action site was mainly skin [17]. The results (Table 3) showed that the β_R/P_ values of PG were 2.28 and 1.63 for the LicA–CP and Gla–CP systems, respectively, which proves that the site of action of the enhancement was mainly the CP matrix for PG. For LicA, the β_R/P_ values of POCC, SP, IPM, and CP were all less than 0.5, demonstrating that skin was the main site of action of the enhancement.

## 4. Discussion

Although we observed an ascending trend for the utilization of enhancers in whitening products for anti-pigmentation, the interaction of whitening compounds, enhancers, and CP or skin in the drug release or permeation process has been neglected, which has resulted in the unreasonable utilization of enhancers and unscientific design of cosmetic formulations. This study systematically demonstrated the quantitative enhancement efficacy of the release and permeation of Gla and LicA by enhancers with different physicochemical parameters, providing a comprehensive understanding of the interaction of drugs, enhancers, and polymer or skin. More importantly, we provided strategies for reasonable selection of enhancers in hydrogel formulations to obtain high drug release and permeation.

Both LicA and Gla showed an anti-melanogenic effect in our previous studies; however, their poor solubility and high partition coefficient affected their formulation design and storage stability. In this work, to ensure the complete dispersion of drug in the CP system, 5% Gla and LicA (*w/w*) were added, respectively, and the XRD and PLM study confirmed this. FT-IR and Raman studies together indicated weak Van der Waals forces interactions present in the Gla–CP and LicA–CP systems. Moreover, the interaction of Gla–CP was significantly higher than that of the LicA–CP binary system due to the better compatibility between the drug and CP. Correspondingly, LicA–CP was expected to possess a higher release percent than that of the Gla–CP due to its easier escape from the hydrogel network. However, the result was contrary to this. Previous studies concluded that the intermolecular force, viscoelasticity, and mesh size of the drug-loaded hydrogels jointly influenced the drug release from hydrogel [4]. The mesh size can be tested by thermal analysis, which was performed to reflect the molecular mobility of the hydrogel and is described by T_g_. A lower T_g_ is indicative of good molecular flexibility and a larger mesh size [26]. Next, the study confirmed that the G′, G″, and mesh size of the hydrogel did not significantly contribute to the drug release percent supported by rheological studies and DSC. In fact, drug release from hydrogel is due to the two processes of hydrogel relaxation and drug diffusion while intermolecular force, viscoelasticity, and the mesh size mainly influence the hydrogel relaxation process [27,28,29]. When diffusion dominates drug release, the drug release percent is primarily inhibited by the drug solubility in receptor fluids. In this work, it was found that Gla had a significantly higher solubility in PEG 400/PBS (20/80, *v/v*) (Table 1), thereby facilitating a higher Gla release percent.

To obtain a higher drug release percent, hydrophilic and hydrophobic enhancers were added. Moreover, the proportion of enhancers was chosen to be 10% to acquire an apparent release and penetration enhancement effect, and to obtain a stronger drug–enhancers–CP or drug–enhancers–skin interaction. Both the LicA and Gla release amount promoted an increase in the MW of the enhancers, which is attributed to the enhanced mesh size induced by the increasing MW. This conclusion is supported by the results showing that molecules with high MW can form a larger pore size in the hydrogel network, leading to a higher drug release amount [3,30]. More importantly, we found that enhancers with high polarizability (α) had higher ER_release_ for both LicA and Gla. The higher polarizability suggests that the drug was more easily polarized by polar molecules, which indicates stronger Van der Waals forces [16]. As a result, enhancers, which had higher polarizability, could link with the drug–CP binary systems to form drug–enhancers–CP ternary systems, or occupied drug–CP sites to form enhancers–CP binary systems. The detailed explanation is discussed below. To further confirm these results, we calculated the E_mix_ and χ values of different drug–enhancers–CP systems and CED of the built amorphous cell systems. Interestingly, th eLicA and Gla release percent showed a good positive correlation with E_mix_ and a negative relationship with CED. Thus, the key intermolecular interaction (Van der Waals forces) was theoretically consistent with the results of the correlation analysis, underscoring the dominant role of interaction forces in hindering drug release.

Next, FT-IR was used to demonstrate the enhancement site of the different enhancers and the enhancement efficacy can be reflected by the displacement degree of –OH and C=O groups. In this work, dry CP rather than aqueous CP was utilized to measure the whole system’s energy. Previous studies revealed that the C=O group show a redshift in CP systems from a dry to a hydrated state, leading to a discrepancy in the interaction between drugs and CP in the two systems. The presence of water reduces the ionic interaction force and increases the H-bonding between the drug and polymers [31,32]. In this work, Van der Waals forces were the dominating forces that controlled the drug release; therefore, dry CP is suitable as an alternative to hydrated CP to measure the interaction in different hydrogel systems. For LicA–CP and Gla–CP systems, POCC and SP generated stronger Van der Waals forces with CP by linking with the –OH group, which inhibited the drug release. However, the addition of CP 90 or NMP did not cause movement of the –OH group. These results proved that –OH of CP was not the enhancement site of drug release, which was different from the –OH enhancement site for loxoprofen release from the PSA matrix in a previous study [17]. The re-crystallization of the drug after the addition of enhancers was also necessary to assess the enhancement mechanism and higher drug re-crystallization indicates a better drug release ability. It was seen that a significantly larger amount of LicA crystals were re-crystallized in the film after the addition of PG or NMP while CP 90 and TP contributed to Gla re-crystallization. This result was consistent with the in vitro release study. It further proved that the drug–CP interaction was destroyed by the enhancers and enhancers–CP interactions occurred.

From the F_P/Q_ value (Table 3), the skin barrier is still the dominating rate-limiting step for Gla and LicA permeation. The amount of Gla retention was significantly higher than LicA retention due to the higher log P of LicA, resulting in better miscibility with skin, which hindered LicA molecules’ penetration into the skin. This result was consistent with previous reports. Usually, permeation enhancers improve the permeation of drug into the skin by disordering the arrangement of lipids and improving the lipid flexibility of SC [11,33]. Moreover, this process is dependent on the physicochemical properties of the enhancers rather than the amount of enhancers [34]. In this work, more LicA and Gla molecules were expected to accumulate in the skin to exert a remarkable anti-melanogenic effect. The seven enhancers chosen could all improve the LicA permeation and retention proportionally. For LicA, enhancers with high log P showed the highest ER_permeation_ and ER_solution retention,_ whereas enhancers with high polarizability facilitated a higher amount of Gla retention. Enhancers with high log P, such as POCC, weakened the LicA–skin interaction and then decreased the interaction forces of the whole LicA–enhancers–skin ternary systems, thereby improving the diffusion of LicA into the skin. Thus, a good linear relation was observed between Q_retention_ (LicA) and CED or E_mix_ after different enhancers were used. However, Gla–skin interaction, which was weaker than LicA–skin, was easier to be destroyed by enhancers with higher polarizability. Enhancers with higher polarizability could also occupy the Gla–skin binding sites to decrease the intermolecular force of the whole system. Taken together, the addition of enhancers disrupted the drug–skin interaction and then reduced the interaction forces of the whole systems, resulting in an improvement of the drug retention or permeation.

FT-IR is an analytical tool used to measure the disorder degree of proteins and lipids in the skin. Porcine skin was used for in vitro skin penetration studies because it possesses a similar epidermal thickness, lipid composition, low frequency impedance, and more importantly, permeability with human skin. Therefore, the V_as_CH_2,_ V_s_CH_2_ and Amide I, Amide II displacement values were not as significant as rat skin [35,36]. A slight movement of the CH_2_ or amide groups results in disarrangement of lipids or distortion of the protein structure. POCC and SP enhanced LicA retention by interacting with the C=O group of the ceramide, thereby a blueshift of the Amide I and Amide II bands was observed. Similarly, C=O was also the main enhancement site for Gla retention by the seven enhancers, indicating enhancers decreased the barrier’s resistance by distorting the protein structure for enhanced LicA and Gla retention. Since Gla showed weaker interaction forces with skin than that of LicA, the seven enhancers all occupied the Gla–skin binding sites and improved Gla accumulation in the skin. CLSM is another tool that was used to confirm the effect of the enhancers on LicA and Gla retention. Higher fluorescence intensity and deeper skin location were indicative of a stronger penetration enhancement effect. This result was also consistent with the in vitro permeation study. Interestingly, the enhancers mainly facilitated drug retention via hair follicle pathways, which was mainly attributed to large hair follicles and a high number of hair follicles in porcine skin [37].

CP hydrogel is a complicated system including CP, water, and drug. Thus, the drug permeation and retention behaviors of hydrogel are not simply a combination of drug release and skin penetration. For LicA, the seven enhancers all improved LicA accumulation in the skin from the solution; however, PG showed the highest ER_hydrogel retention_ and ER_release_ for LicA, indicating that both the drug release and skin permeation processes limited LicA’s permeability. Interestingly, the seven enhancers all showed a significant enhancement effect on Gla retention from hydrogel, which was different from the enhanced effect of Gla retention by CP, POCC, and PG in solution. These results further indicate that the main rate-limiting of Gla’s penetration is its skin permeability.

## 5. Conclusions

In this work, a systematic approach was established to evaluate the enhanced release and retention of whitening agents from CP hydrogel in the presence of enhancers based on interactions among the drug, enhancers, and CP or skin. ER_release_, ER_permeation_, ER_com_, ER_solution retention_, and ER_hydrogel retention_ were utilized to evaluate the quantitative enhanced effect, and β_R/P_ was calculated to evaluate the enhancement action sites of the enhancers. We found that the release and retention enhancement effect were closely related to the structures of the enhancers. Gla–CP hydrogel showed higher drug release and retention ability than LicA–CP, which was attributed to the higher solubility in medium and better miscibility with skin of Gla than that of LicA. Enhancers with higher MW and lower polarizability showed a higher ER_release_ for both LicA and Gla, whereas enhancers with higher log P and polarizability displayed a higher ER_solution retention_ for LicA and Gla. More importantly, Van der Waals forces among the drug, enhancers and CP showed a negative correlation with the drug release percent, and intermolecular interaction between the drug, enhancers, and skin also showed a linear decreasing effect on drug retention. Additionally, the C=O group of the ceramide was the enhancement site for drug permeation by the enhancers. Consequently, TP and PG, and the seven enhancers showed a higher ER_hydrogel retention_ for LicA–CP and Gla–CP respectively. Taken together, the conclusions provide a strategy for reasonable utilization of enhancers and formulation optimization in whitening topical hydrogels.

## Figures and Tables

**Table 1:** The physicochemical parameters of different drugs and enhancers.

Drug/Enhancers	Molecular Weight (Da)	Log P	Solubility in 20% PEG400 (*v/v*, µg/mL)	H Bond Donor	H Bond Acceptor	Polarizability	Polar Surface Area (Å)
LicA	338.40	4.95	25.15	2	4	39.8	66.8
Gla	324.40	4.26	121.47	2	4	36.1	58.9
CP 90	203.30	4.97	-	-	-	33.5	47.0
IPM	270.40	4.61	-	-	-	32.7	26.0
NMP	99.10	−0.38	-	-	-	10.6	20.0
PG	76.09	−0.90	-	-	-	7.52	40.5
POCC	726.90	6.21	-	-	-	74.8	269.0
SP	428.60	6.06	-	-	-	47.3	96.0
TP	134.20	−1.08	-	-	-	13.8	39.0

**Table 2:** The E_mix_, χ, and CED of different Gla (LicA)–enhancers–CP systems.

	χ (kcal/mol)	E_mix_ (kcal/mol)	CED (kcal/mol)
LicA-CP	20.52	12.15	-
Gla-CP	13.00	7.70	-
LicA-CP 90-CP	6.97	4.13	2.57 × 10^9^
LicA-IPM-CP	6.56	3.89	2.38 × 10^9^
LicA-NMP-CP	10.87	6.44	2.21 × 10^9^
LicA-PG-CP	19.48	11.54	2.15 × 10^9^
LicA-POCC-CP	−7.15	−4.23	2.40 × 10^9^
LicA-SP-CP	−1.77	−1.05	2.34 × 10^9^
LicA-TP-CP	9.35	5.54	2.40 × 10^9^
Gla-CP 90-CP	2.45	1.45	2.60 × 10^9^
Gla-IPM-CP	3.01	1.78	2.64 × 10^9^
Gla-NMP-CP	6.50	3.85	2.33 × 10^9^
Gla-PG-CP	8.85	5.24	2.36 × 10^9^
Gla-POCC-CP	2.57	1.52	2.68 × 10^9^
Gla-SP-CP	6.37	3.77	2.38 × 10^9^
Gla-TP-CP	6.34	3.76	2.43 × 10^9^

**Table 3:** The enhancement efficacy parameters of enhancers in the drug release and skin penetration process.

	ER_release_	ER_permeation_	ER_com_	β_R/P_	ER_solution retention_	ER_hydrogel retention_	F_P/Q_
LicA-CP 90	0.89	2.72	1.18	0.33	2.70	1.13	0.015
LicA-IPM	1.05	2.91	0.00	0.36	3.30	0.68	0.00
LicA-NMP	1.21	1.08	0.00	1.12	0.54	0.76	0.00
LicA-PG	1.25	0.55	1.10	2.28	1.23	1.82	0.0099
LicA-POCC	0.89	3.09	1.46	0.29	3.28	0.65	0.018
LicA-SP	1.02	2.77	1.12	0.37	3.78	0.99	0.012
LicA-TP	1.00	1.50	1.92	0.67	1.44	1.23	0.022
Gla-CP 90	1.07	2.93	1.40	0.36	2.11	1.39	0.022
Gla-IPM	1.08	1.15	0.86	0.94	1.49	1.51	0.014
Gla-NMP	1.14	2.20	0.78	0.52	1.47	1.28	0.012
Gla-PG	1.11	0.68	1.28	1.63	1.31	1.11	0.020
Gla-POCC	1.06	2.16	2.05	0.49	1.28	1.79	0.033
Gla-SP	1.14	1.15	1.14	0.99	1.04	1.90	0.017
Gla-TP	1.15	1.38	0.72	0.83	1.51	1.14	0.011

**Table 4:** Molecular docking and molecular dynamics (MD) simulation results of Gla (LicA)–enhancers–skin systems.

	χ (kcal/mol)	E_mix_ (kcal/mol)	CED (kcal/mol)
LicA-CP 90-Skin	27.54	16.31	1.49 × 10^9^
LicA-IPM-Skin	24.82	14.70	1.37 × 10^9^
LicA-NMP-Skin	39.04	23.12	1.59 × 10^9^
LicA-PG-Skin	48.50	28.72	1.62 × 10^9^
LicA-POCC-Skin	17.13	10.14	1.41 × 10^9^
LicA-SP-Skin	16.74	9.92	1.35 × 10^9^
LicA-TP-Skin	42.52	25.18	1.49 × 10^9^
Gla-CP 90-Skin	10.82	6.41	1.30 × 10^9^
Gla-IPM-Skin	6.97	4.13	1.39 × 10^9^
Gla-NMP-Skin	28.00	16.58	1.46 × 10^9^
Gla-PG-Skin	31.06	18.40	1.54 × 10^9^
Gla-POCC-Skin	14.07	8.33	1.45 × 10^9^
Gla-SP-Skin	1.44	0.86	1.54 × 10^9^
Gla-TP-Skin	21.41	12.68	1.47 × 10^9^

## Data Availability

Not applicable.

## Supplementary Materials

The following are available online at https://www.mdpi.com/article/10.3390/pharmaceutics14020262/s1, Methods S1: The HPLC methods of LicA and Gla, Figure S1: (a) DSC curves of different hydrogels; (b) Flow characterization of the hydrogels (n = 3); (c) In vitro drug release profiles of LicA-CP hydrogel when enhancers were added (n = 3); (d) In vitro drug release profiles of LicA-CP hydrogel when enhancers were added (n = 3), Figure S2: Conformation of LicA-enhancers-CP and Gla-enhancers-CP ternary systems, Figure S3: The snapshots of LicA (Gla)-enhancers-CP systems at the end of the MD. (Drug: Ball and stick model; Enhancers: CPK model), Figure S4: (a) FT-IR spectra (C=O group) of LicA-enhancers-CP systems; (b) FT-IR spectra (C=O group) of Gla-enhancers-CP systems; (c) PLM images of drug-CP films after different enhancers were added, Figure S5: (a) FT-IR spectra (CH_2_- group) of LicA-enhancers-skin systems; (b) FT-IR spectra (CH2-group) of Gla-enhancers-skin systems; (c) CLSM images of penetration depth and fluorescence intensity LicA and C6 in porcine skin treated by enhancers (bar = 100 μm), Figure S6: Conformations of LicA-enhancers-skin and Gla-enhancers-skin ternary systems, Figure S7: The snapshots of LicA (Gla)-enhancers-skin systems at the end of the MD. (Drug: Ball and stick model; Enhancers: CPK model).
