# Supplementary material for: Quantitative Structure-Activity Relationship of Enhancers of Licochalcone A and Glabridin Release and Permeation Enhancement from Carbomer Hydrogel
Source: Pharmaceutics. 2022 Jan 22;14(2):262. doi: 10.3390/pharmaceutics14020262 (PMC8878673; doi:10.3390/pharmaceutics14020262)
Supplement: Supplementary file 1 [file pharmaceutics-14-00262-s001.zip › pharmaceutics-1547570-SI.pdf]

pharmaceutics

Supplementary Materials: Quantitative Structure–Activity Relationship of Enhancers of Licochalcone A and Glabridin Release and Permeation Enhancement from Carbomer Hydrogel

Zhuxian Wang, Yaqi Xue, Zhaoming Zhu, Yi Hu, Quanfu Zeng, Yufan Wu, Yuan Wang, Chunyan Shen, Cuiping Jiang, Li Liu, Hongxia Zhu and Qiang Liu

Methods S1 The HPLC methods of LicA and Gla

The HPLC method of LicA was showed as follows: The samples were analyzed by HPLC (Agilent 1260, USA)equipped with C18 column and DAD detector. The mobile phase consisted of combinations of A (methanol), B (acetonitrile) and C (0.1% phosphate in water, *v*/*v*) at a flow rate of 1.0 mL/min with an elution as follows: 0–13 min, 20% A and 45% B. The detection wavelength was set at 300 nm with a retention time of 8.36 min. The calibration curve was linear over the range 0.01–100 μg/mL (r^2 = 0.9992). The injection volume was 20 μL. For Gla, the detection wavelength was set at 230 nm with a retention time of 12.44 min, and the calibration curve was linear over the range 0.01–100 μg/mL (r^2 = 0.9995)

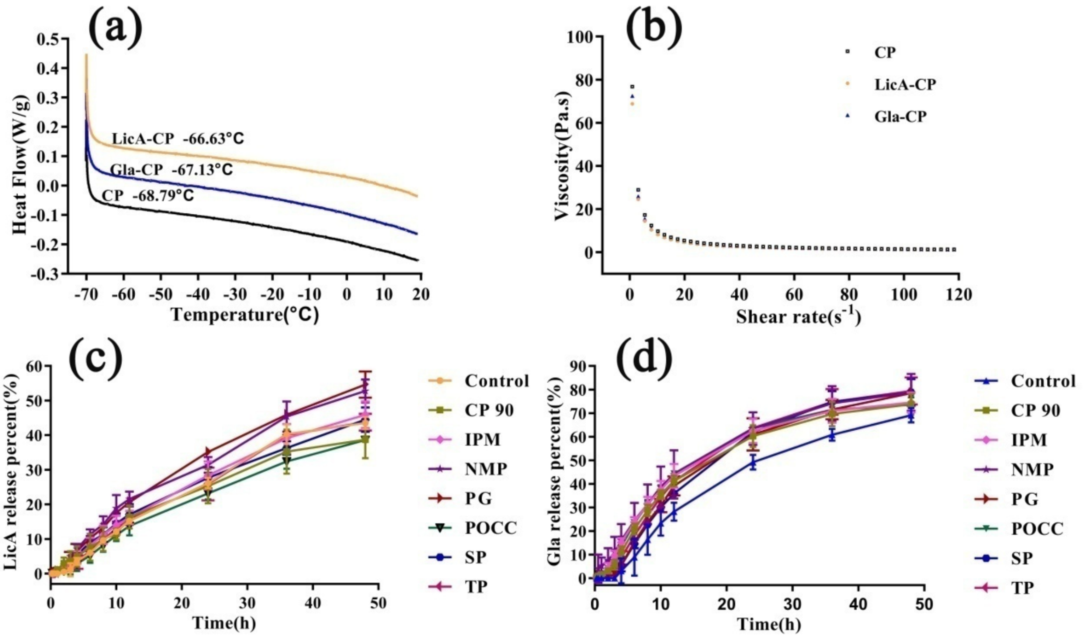

Figure S1. (**a**) DSC curves of different hydrogels; (**b**) Flow characterization of the hydrogels (n = 3); (**c**) In vitro drug release profiles of LicA-CP hydrogel when enhancers were added (n = 3); (**d**) In vitro drug release profiles of LicA-CP hydrogel when enhancers were added (n = 3).

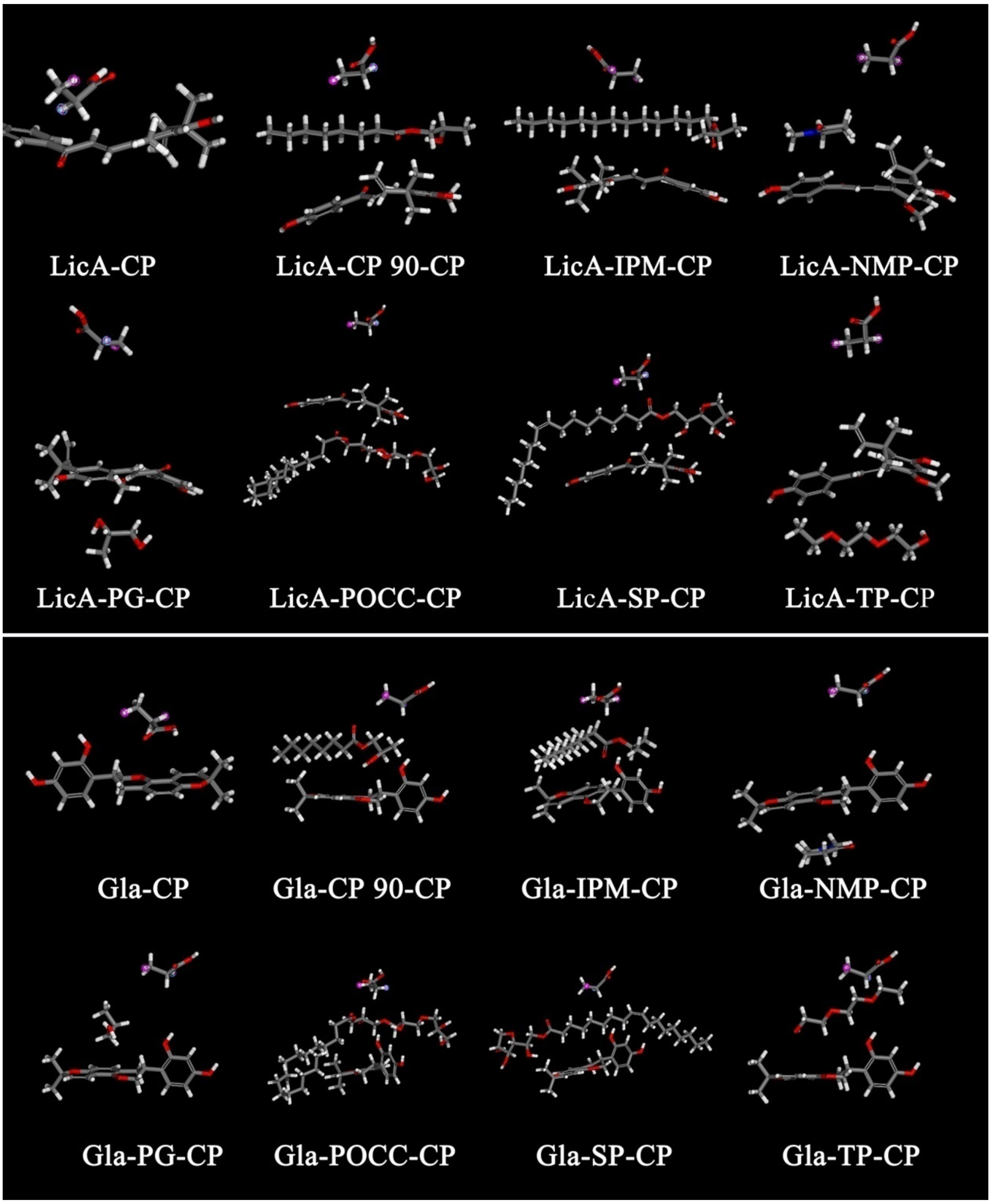

Figure S2. Conformation of LicA-enhancers-CP and Gla-enhancers-CP ternary systems.

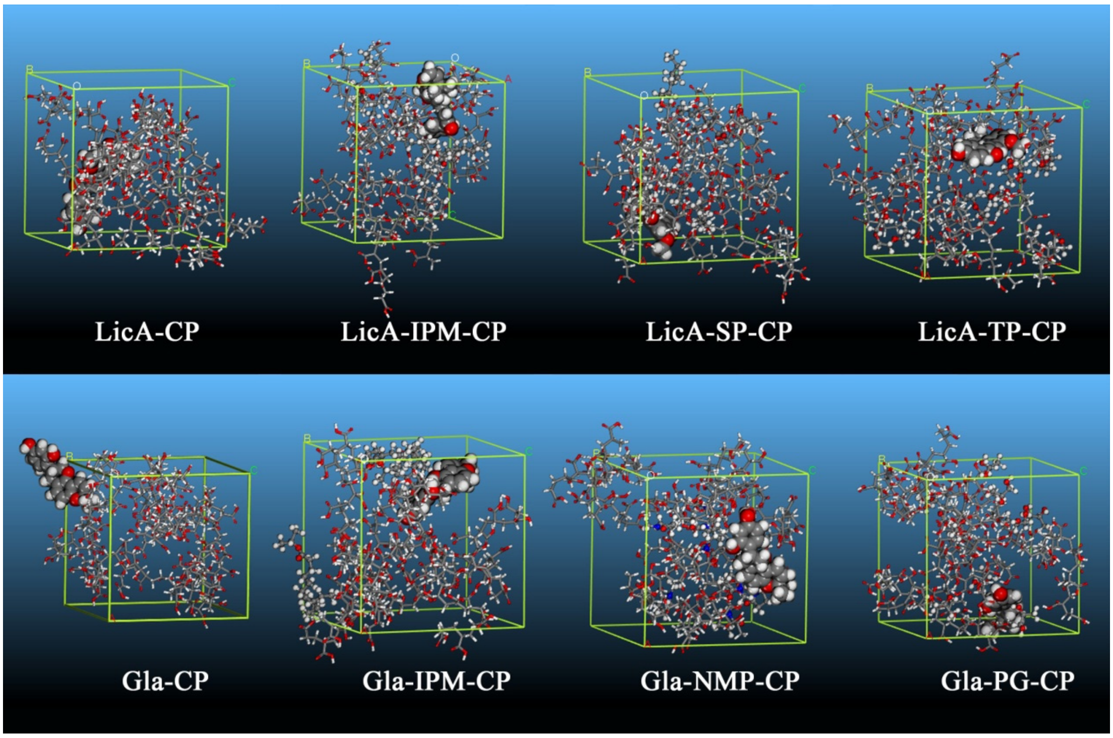

Figure S3. The snapshots of LicA (Gla)-enhancers-CP systems at the end of the MD. (Drug: Ball and stick model; Enhancers: CPK model).

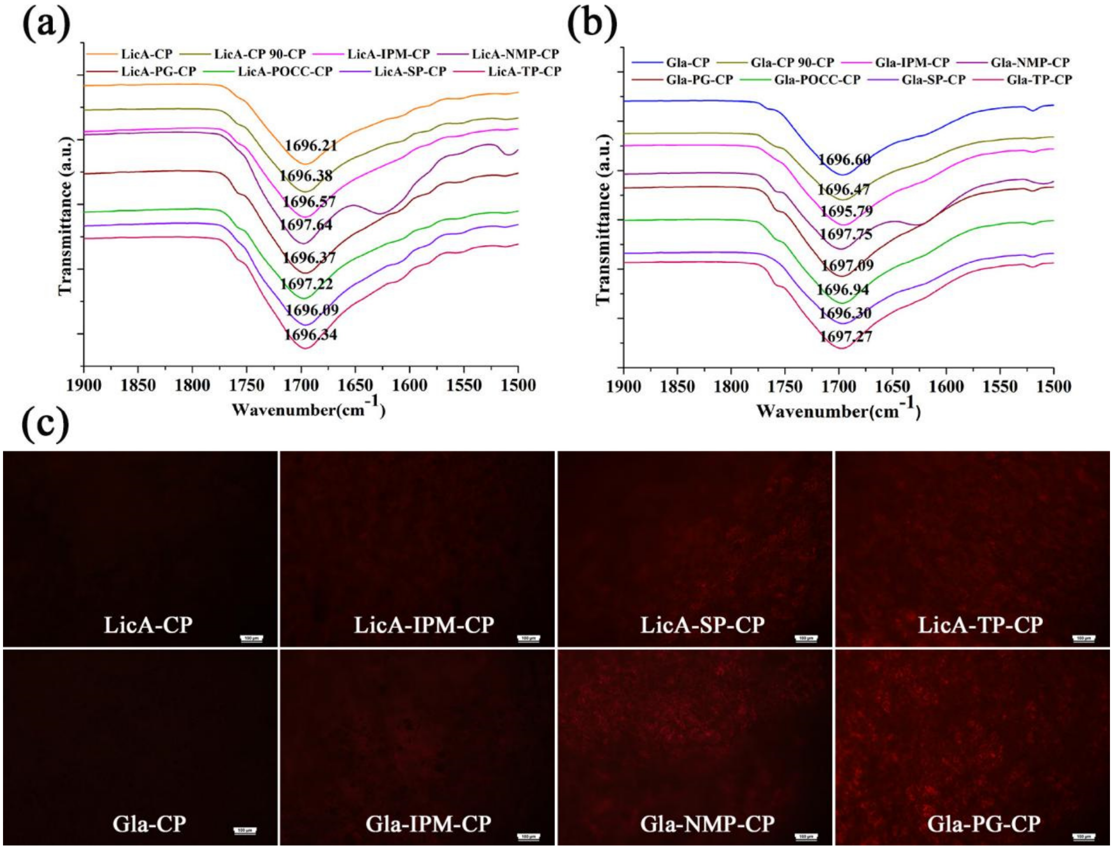

Figure S4. (**a**) FT-IR spectra (C=O group) of LicA-enhancers-CP systems; (**b**) FT-IR spectra (C=O group) of Gla-enhancers-CP systems; (**c**) PLM images of drug-CP films after different enhancers were added.

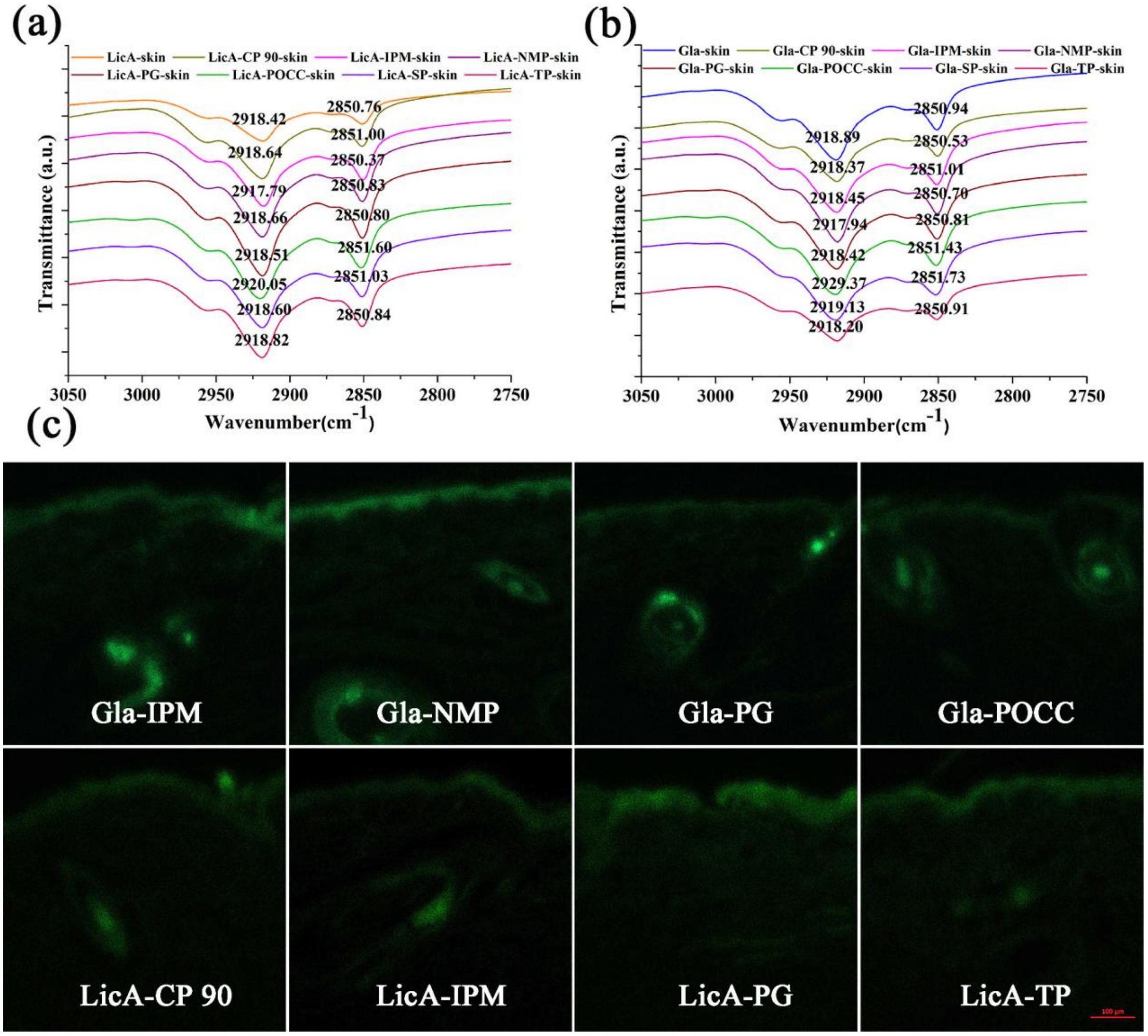

Figure S5. (**a**) FT-IR spectra (CH_2-group) of LicA-enhancers-skin systems; (**b**) FT-IR spectra (CH_2-group) of Gla-enhancers-skin systems; (**c**) CLSM images of penetration depth and fluorescence intensity LicA and C6 in porcine skin treated by enhancers (bar = 100 μm).

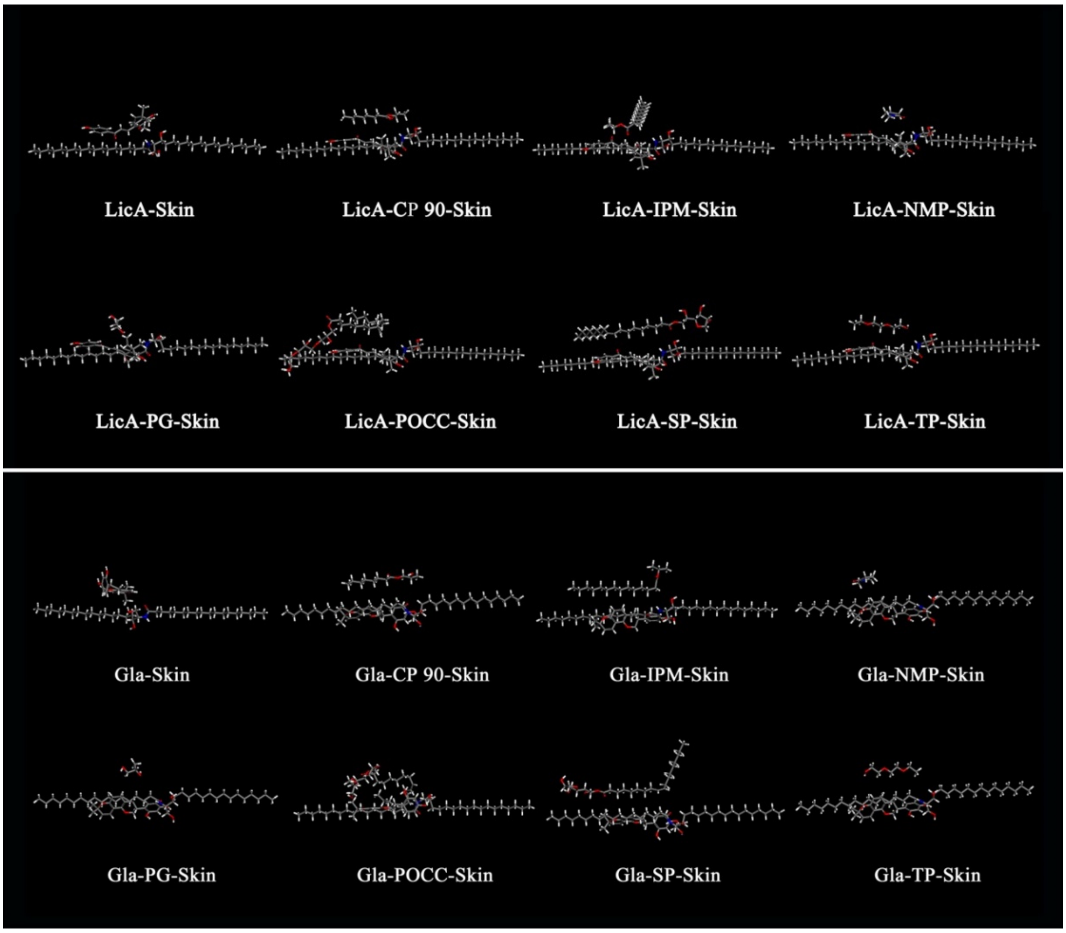

Figure S6. Conformations of LicA-enhancers-skin and Gla-enhancers-skin ternary systems.

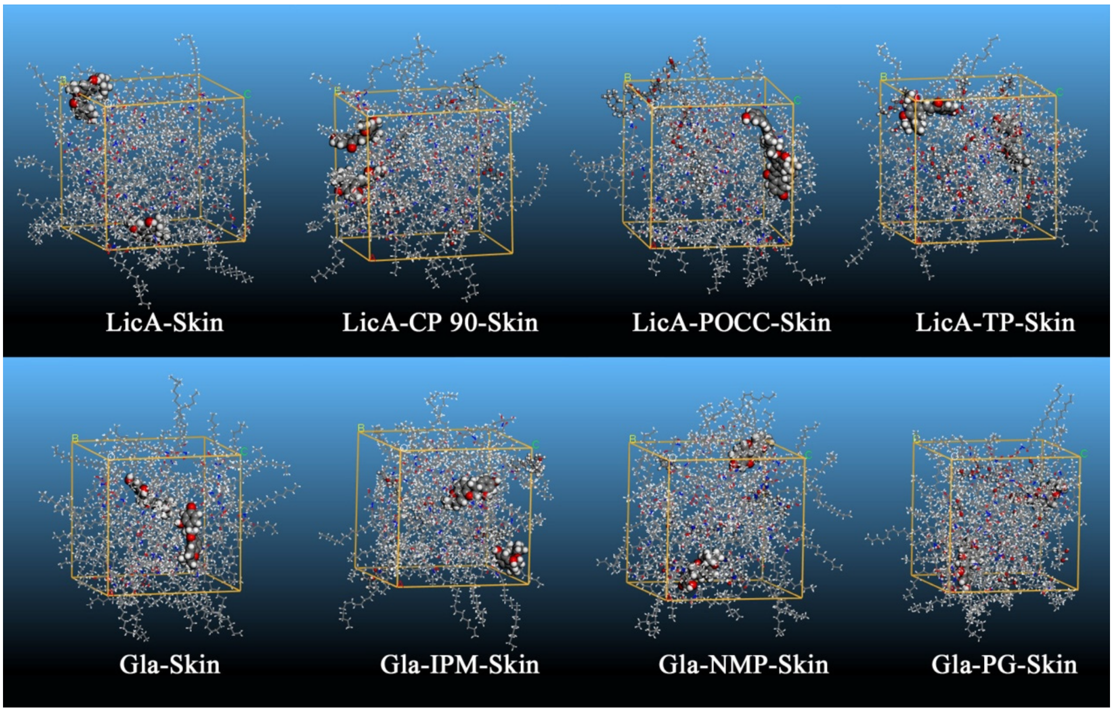

Figure S7. The snapshots of LicA (Gla)-enhancers-skin systems at the end of the MD. (Drug: Ball and stick model; Enhancers: CPK model).
